# Comparative Analysis of the Tolerance of Young and Old Kidneys to Injury in a Rat Model of Reversible Ureteral Obstruction

**DOI:** 10.3390/antiox14101219

**Published:** 2025-10-10

**Authors:** Polina A. Abramicheva, Ilya A. Sokolov, Vasily N. Manskikh, Nadezda V. Andrianova, Dmitry S. Semenovich, Ljubava D. Zorova, Irina B. Pevzner, Egor Y. Plotnikov

**Affiliations:** 1 A.N. Belozersky Institute of Physico-Chemical Biology, M.V. Lomonosov Moscow State University, 119992 Moscow, Russiamanskikh@mail.ru (V.N.M.); andrianova@belozersky.msu.ru (N.V.A.); dima-chem1@rambler.ru (D.S.S.); ljuzor@belozersky.msu.ru (L.D.Z.); pevzner_ib@belozersky.msu.ru (I.B.P.); 2 Faculty of Biology, M.V. Lomonosov Moscow State University, 119991 Moscow, Russia; 3 V.I. Kulakov National Medical Research Center of Obstetrics, Gynecology, and Perinatology, 117997 Moscow, Russia

**Keywords:** reversal UUO, obstructive nephropathy, aging, fibrosis, kidney, mitochondrial dysfunction

## Abstract

Obstructive nephropathy is a common clinical condition caused by urinary retention. After urine flow is restored, kidney function is recovered. However, the effectiveness of this process can be influenced by many factors, including the age of the patient. In this study, we analyzed the following parameters in young and old rats subjected to a 3-day reversible unilateral ureteral obstruction (R-UUO): AKI severity, renal tissue proliferation and histology, inflammatory and fibrosis marker expression, as well as autophagosomal-lysosomal and mitochondrial function. Compared to old rats, young animals exhibited more pronounced renal tissue proliferation and higher expression of profibrotic markers (*Col1a1*, *Fn1*, *Tgfb1*, *MMP2*), but diminished expression of pro-inflammatory markers (*Il1b*, *Tnfa*, *Cd32*) in response to R-UUO. Additionally, young rats showed more pronounced activity of autophagy, as indicated by increased beclin-1 levels. R-UUO induced severe damage to the mitochondrial respiratory chain in old animals, as indicated by reduced complex I, IV, cytochrome c, VDAC protein levels, and impaired mitochondrial biogenesis (associated with decreased *Pgc1a* mRNA expression). Thus, we demonstrated that despite restored urine outflow, kidneys exhibited autophagy activation, inflammatory response, and mitochondrial dysfunction after R-UUO. Negative alterations in the kidney were age-dependent indicating necessity for therapeutic strategies optimization for patients of different ages.

## 1. Introduction

Acute kidney injury (AKI) is a syndrome characterized by a rapid decline in kidney function. It is marked by a significant decrease in the glomerular filtration rate, the death of renal tubules and glomerular cells, and mitochondrial dysfunction. AKI occurs in 10–15% of patients admitted to general medicine wards and in over 50% of intensive care unit patients [1].

Risk factors for AKI include sepsis, diabetes mellitus, genetic predisposition, urolithiasis, kidney transplantation, and advanced age [2]. Without appropriate treatment, AKI can progress to chronic kidney disease (CKD). 5–10% of AKI cases are associated with the development of obstructive nephropathy [3]. This pathology is well-reproduced by the experimental model of unilateral ureteral obstruction (UUO). A modification of this approach, the reversible UUO model (R-UUO), involves releasing the ureteral clamp after a defined period [4,5]. In clinical practice, ureteral obstruction is often transient and typically resolves spontaneously (for example, when stones pass through the ureter (nephrolithiasis)). Consequently, the R-UUO model is considered more clinically relevant. It provides a valuable tool for investigating the mechanisms of renal injury and for analyzing the processes of functional and structural recovery following the relief of obstruction.

Susceptibility to acute and chronic kidney injury is age-dependent [6,7,8]. The majority of hospitalized patients with AKI are over 65 years old [9], a similar age distribution (~60%) is seen in obstructive nephropathy [10,11]. Nonetheless, most therapeutic strategies for renal damage are focused on young patients. Consequently, interventions that prove effective in young animal models often fail to confer a protective effect in older organisms [12,13].

We hypothesized that renal susceptibility to reversible unilateral ureteral obstruction is age-dependent and that aging alters the profile of injury, inflammation, oxidative stress, autophagy, and regenerative response. This study performed a comparative analysis of renal tolerance to R-UUO-induced AKI in young and aged animals. We assessed AKI severity parameters, evaluated renal tissue proliferative capacity and injury-induced histological alterations, the expression of inflammatory markers in renal tissue, and the activity of the autophagosomal-lysosomal system along with mitochondrial function in both young and old rats.

## 2. Materials and Methods

### 2.1. Animals

Experiments were performed on male outbred Wistar rats of two age groups: young (3–4 months old, 300–400 g) and old (21–24 months old, 550–700 g). The study was conducted according to the guidelines of the Declaration of Helsinki, and approved by the A.N. Belozersky Institute of Physico-Chemical Biology Lomonosov Moscow State University: Protocol № 008-3/03/2024 from 11 March 2024. All procedures were executed in accordance with the “Animal Research: Reporting of In Vivo Experiments” (ARRIVE) guidelines.

### 2.2. Experimental Design

**Figure 1:**
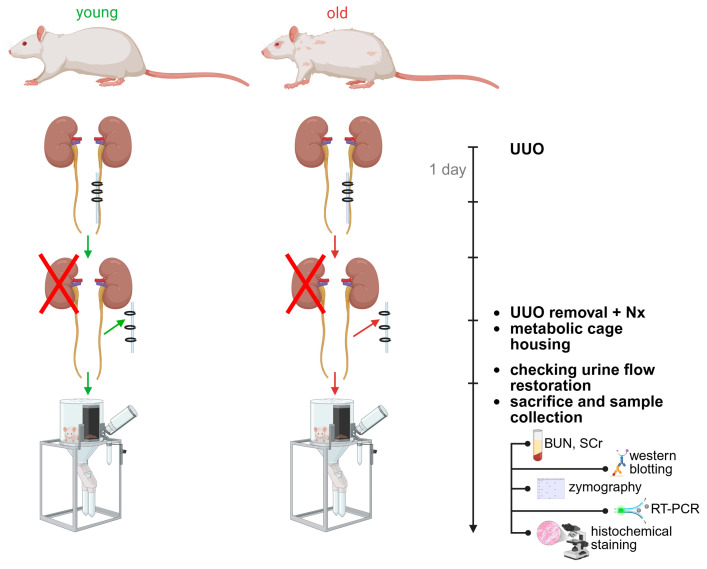
Experimental design and sampling. Abbreviations: BUN—blood urea nitrogen, Nx—contralateral nephrectomy, RT-PCR—real-time PCR, SCr—serum creatinine, UUO—unilateral ureteral obstruction. Created in https://BioRender.com (accessed on 7 October 2025).

Young and old animals were divided into 2 groups: intact (Int), with 3-day reversible UUO followed by contralateral nephrectomy (R-UUO). The 3-day timepoint in the UUO model is well-established in the literature to study early tubular injury and apoptosis, peak inflammatory cell infiltration, initiation of profibrotic signaling and characterized by absence of mature fibrosis [14,15,16,17]. Rats were anesthetized with isoflurane and placed supine on a heating pad. For reversible obstruction, a modified UUO technique was used: a ~0.5 cm silicone tube was ligated around the ureter with 2-0 vicryl to compress the ureter while avoiding injury. Three days post-operation, the surgical sutures were removed, and the silicone tube was carefully extracted to avoid ureteral injury following contralateral nephrectomy. To verify restored urine flow through the previously obstructed ureter, the animals were housed in metabolic cages for 24 h for urine collection. Following this, they were euthanized for sample collection. The left kidneys (with obstructed ureters) were excised, decapsulated, and sectioned. The upper pole was fixed in 10% buffered formalin, while the remaining tissue was homogenized in ice-cold RIPA lysis buffer (Millipore, Temecula, CA, USA) supplemented with 1 mM phenylmethylsulfonyl fluoride (PMSF) (Thermo Fisher Scientific, Carlsbad, CA, USA) and a protease inhibitor cocktail “cOmplete™ ULTRA tablets” (Roche Diagnostics, Meylan, France) for Western blotting, or in PBS buffer for zymography. RT-PCR analysis was performed on tissue from the lower pole of the left kidney. Blood samples were collected for blood urea nitrogen (BUN) and serum creatinine (SCr) measurements (Figure 1).

### 2.3. Serum Analysis

Blood samples were collected from the carotid artery to measure BUN and SCr levels. After clotting for 15 min at room temperature, samples were centrifuged at 2000× *g* for 5 min at 4 °C to remove the clot. Serum BUN and SCr concentrations were then determined using an AU480 Chemistry System (Beckman Coulter, Brea, CA, USA).

### 2.4. Western Blotting

Western blot analysis was performed under denaturing conditions using standard SDS-PAGE techniques. Total protein content was measured using a bicinchoninic acid (BCA) assay kit (Sigma Aldrich, St. Louis, MO, USA). Samples (10 μg protein per well) were separated on a 12% SDS-PAGE gel and transferred to a polyvinylidene fluoride (PVDF) membrane (Amersham Pharmacia Biotech, Buckinghamshire, UK) using the Trans Blot Turbo Transfer System (Bio-Rad, Laboratories Inc., Hercules, CA, USA). Membranes were blocked for 45 min at room temperature with 5% nonfat milk in TBST (TBS + 0.1% Tween-20 (Helicon, Moscow, Russia)) and then washed three times. Subsequently, they were incubated with primary antibodies diluted in 0.1% bovine serum albumin (BSA), against alpha-smooth muscle actin (α-SMA) 1:1000 (ab5694, Rb, Abcam, Cambridge, UK), proliferating cell nuclear antigen (PCNA) 1:1000 (13110, Rb, Cell Signaling, Boston, MA, USA), Acetyl-Histone H3 (Lys9) 1:1000 (9649, Rb, Cell Signaling, Boston, MA, USA), beclin-1 1:1000 (3495, Rb, Cell Signaling, Boston, MA, USA), Sequestosome-1 (SQSTM1)/p62 1:1000 (5114, Rb, Cell Signaling, Boston, MA, USA), complex IV 1:1000 (ab109863, Ms, Abcam, Cambridge, UK), complex I 1:1000 (A21344, Ms, Thermofisher Scientific, Carlsbad, CA, USA), cytochrome c (cyt c) 1:1000 (556433, Ms, BD Biosciences, San Jose, CA, USA), ATP synthase subunit b 1:1000 (MABS1304, Ms, Millipore, St. Louis, MO, USA), voltage-dependent anion-selective channel (VDAC) 1:1000 (4866, Rb, Cell Signaling, Boston, MA, USA), and β-actin 1:2000 (A2228, Ms, Sigma-Aldrich, St. Louis, MO, USA) at 4 °C overnight. Membranes were washed and incubated for 60 min at 37 °C with horseradish peroxidase conjugated goat anti-rabbit or anti-mouse IgG antibodies (IMTEK, Moscow, Russia) at a 1:5000 dilution. After washing, protein bands were visualized by incubating membranes with Advansta ECL Bright chemiluminescence kit (Advansta, San Jose, CA, USA) for 5 min and capturing the chemiluminescent signal with ChemiDoc MP Imaging System (Bio-Rad, Laboratories, Inc., Hercules, CA, USA). Band luminescence intensity was quantified using ImageLab 6.0.1 software (Bio-Rad, Laboratories Inc., Hercules, CA, USA). Band densities were normalized to β-actin.

### 2.5. Zymography

Kidneys were homogenized in PBS containing 1 mM phenylmethylsulfonyl fluoride (PMSF; Thermo Fisher Scientific, Rockford, IL, USA). Protein content was measured using a commercial bicinchoninic acid (BCA) assay kit (Sigma, St. Louis, MO, USA). Homogenized samples (20 μg total protein per well) were separated on a 10% SDS-PAGE gel co-polymerized with 2 mg/mL gelatin (Sigma-Aldrich, Darmstadt, Germany). Following electrophoresis, the gel was incubated in the renaturation buffer (Invitrogen, Novex, Carlsbad, CA, USA) and then activated in the development buffer (Invitrogen, Carlsbad, CA, USA). Gel was stained with 0.25% Coomassie Brilliant Blue R-250 for 1 h, followed by destaining until clear gelatinolytic bands appeared against the blue background. Matrix metalloproteinase (MMP) activity, visualized as zones of gelatin degradation, was quantified using densitometric analysis with ImageLab 6.0.1 software (Bio-Rad, Laboratories Inc., Hercules, CA, USA).

### 2.6. RT-PCR

The mRNA expression levels of fibrosis-related genes (*Col1a1*, *Fn1*, *Tgfb1*, *Mmp2*), inflammatory response genes (*Il1b*, *Tnfa*,), total markers of leukocytes (*Cd45*), neutrophils (*Cxcl1*), macrophages (*Cd68*), NK-cells (natural killers) and monocytes (*Cd32*), M1- and M2-macrophages (*Tlr2*, and *Cd206*, respectively) as well as oxidative stress markers (*Nrf2, Gpx1*, *Pgc1a*) were quantified in young and old rats following R-UUO. The 60S acidic ribosomal protein (*Rplp0*) was used as the housekeeping gene for normalization. Total RNA was extracted from renal tissue samples using TRIzol reagent (Thermo Fisher Scientific, Carlsbad, CA, USA). The aqueous phase, containing nucleic acids, was precipitated with 75% ethanol following homogenization and chloroform centrifugation. RNA from renal tissue was further purified, including a DNase treatment step using the RNeasy Mini Kit (QIAGEN, Hilden, Germany) and reverse-transcribed into cDNA using the MMLV RT Kit (Evrogen, Moscow, Russia) according to the manufacturer’s guidelines. Real-time PCR was performed using the Bio-Rad CFX96 Real-Time PCR System (Bio-Rad, Hercules, CA, USA) and 5× qPCRmix-HS master mix (Evrogen, Moscow, Russia). Primer design was performed using the cloud-based Benchling platform (San Francisco, CA, USA) and Primer-BLAST (NCBI/NLM, Bethesda, Montgomery, MD, USA). All oligonucleotides were subsequently synthesized by DNA-synthesis (Moscow, Russia). Primer sequences are provided in Table S1.

### 2.7. Histochemical Analysis

To evaluate scar tissue formation, renal tubule dilation, and age-related changes in the kidney, paraformaldehyde-fixed kidneys were embedded in paraffin and sectioned at 4 μm. For periodic acid–Schiff (PAS) staining, sections were oxidized with 0.5% periodic acid for 5 min, rinsed in distilled water, and then stained with Schiff reagent for 15 min. After staining, sections were rinsed under running tap water for 5 min, and nuclei were counterstained with alum hematoxylin. All stained kidney sections were examined using the CELENA^®^ X High Content Imaging System (Anyang-si, Gyeonggi-do, Republic of Korea).

### 2.8. Statistical Analysis

Statistical analyses were performed using two-way ANOVA with Dunnett post hoc test based on their distribution normality (Shapiro–Wilk normality test) in GraphPad Prism 10 (GraphPad Software Inc., La Jolla, CA, USA). To identify and exclude outliers, we used Grubbs’ and ROUT tests. All data are presented as mean ± SEM.

## 3. Results

### 3.1. The Severity of Renal Injury Induced by R-UUO

**Figure 2:**
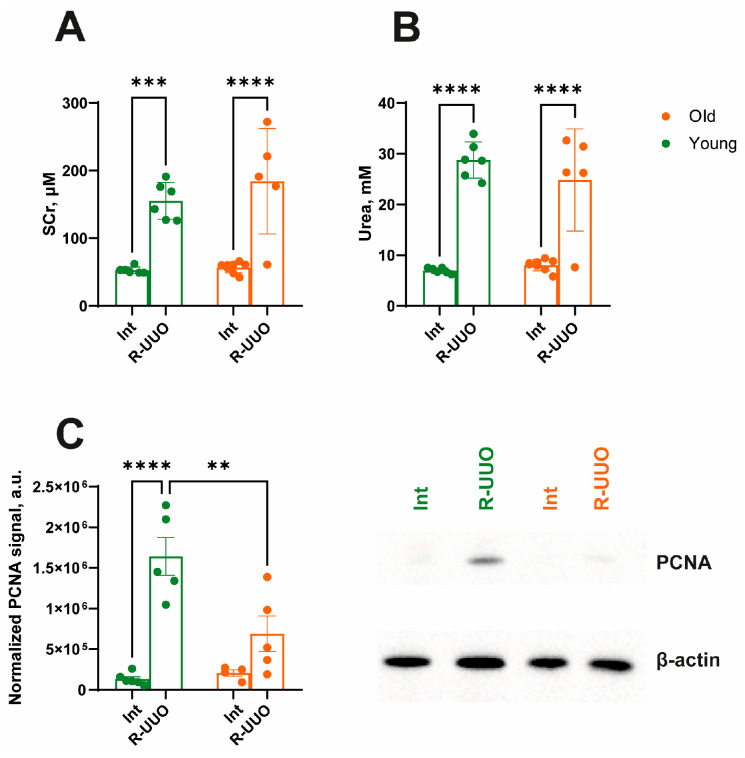
Assessment of kidney injury after R-UUO in young and old rats. Renal failure measured by SCr (**A**) and BUN (**B**) 24 h after urine flow restoration. (**C**) The increase of proliferation evaluated by PCNA levels in the kidney. The data for young rats are shown in green, and the data for old rats are shown in orange. ** *p* < 0.01, *** *p* < 0.001, **** *p* < 0.0001 statistically significant differences (two-way ANOVA, Dunnett’s post hoc test). The examples shown are representative of at least 4 independent experiments.

Conventional markers of kidney function, SCr and BUN, were elevated after R-UUO induction in both young and old rats (Figure 2A,B), indicating a decline in glomerular filtration rate (GFR) and enhanced protein catabolism [18,19]. To assess the regenerative and proliferative potential of the kidney following R-UUO-induced injury, we analyzed PCNA levels. We showed that PCNA expression induced by R-UUO was significantly higher in young animals compared to old ones (Figure 2C).

### 3.2. R-UUO Induces More Pronounced Fibrosis Marker Expression in Young Rats Compared to Old

**Figure 3:**
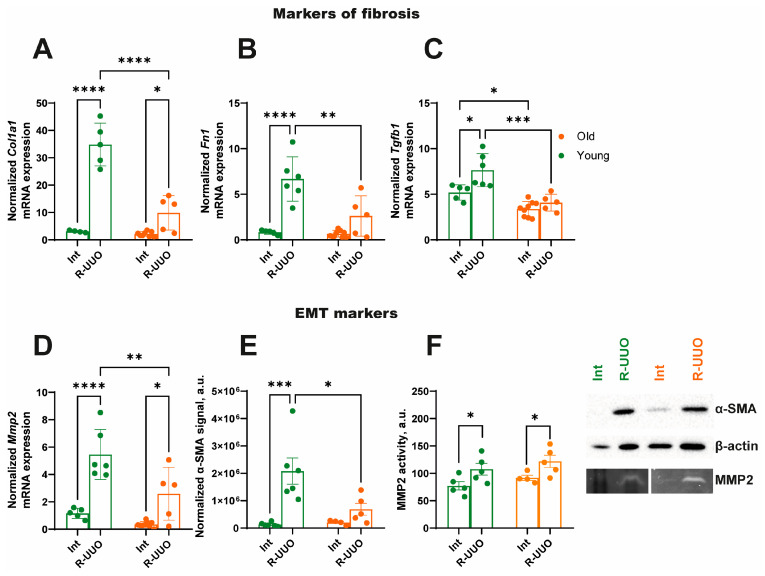
Expression of markers of fibrosis and epithelial-to-mesenchymal transition (**A**–**D**), protein level of α-SMA (**E**), and activity of MMP2 (**F**) in young and old rats. The data for young rats are shown in green, and the data for old rats are shown in orange. * *p* < 0.05, ** *p* < 0.01, *** *p* < 0.001, **** *p* < 0.0001 statistically significant differences (two-way ANOVA, Dunnett’s post hoc test). The examples shown are representative of at least 4 independent experiments.

We demonstrated that R-UUO stimulated the expression of fibrosis and epithelial-to-mesenchymal transition marker genes, with a more pronounced response in young compared to old animals (Figure 3A–D). A similar expression pattern was observed for the fibrosis marker α-SMA (Figure 3E). In contrast, while the activity of matrix metalloproteinase 2 (MMP2)—a known trigger of AKI [20,21]—increased in response to renal damage, it showed no significant age-related differences (Figure 3F).

### 3.3. R-UUO-Induced Histopathological Changes in the Kidney

**Figure 4:**
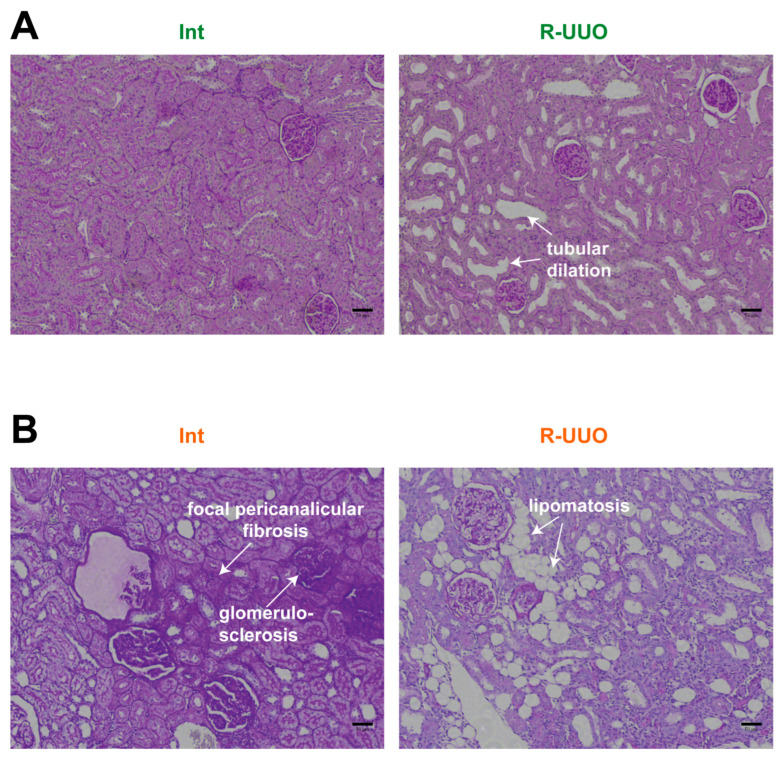
Assessment of renal morphology in young and old rats following reversible obstruction. Analysis of renal injury and age-related alterations in PAS-stained kidney sections of young (**A**) and old (**B**) rats. Green indicates microscopic images from kidneys of young rats, and orange indicates those from old rat’ kidneys. Scale bar: 50 µm.

As a well-established model of tubulointerstitial fibrosis [14], UUO induces significant histological alterations in kidney morphology. We investigated how aging modulates these changes in intact kidneys and following R-UUO. Although urine flow was restored after blockage removal, young rats in the R-UUO group still showed marked tubular dilation (Figure 4A). In old rats, severe tubular dilation was accompanied by additional age-related pathologies indicative of declining renal function. These included focal pericanalicular fibrosis (deposition of extracellular matrix around tubules), glomerulosclerosis, and lipomatosis—the progressive replacement of renal parenchyma with adipose tissue [22]) (Figure 4B).

### 3.4. Age-Related Differences in Immune Cell and Cytokine Marker Expression

**Figure 5:**
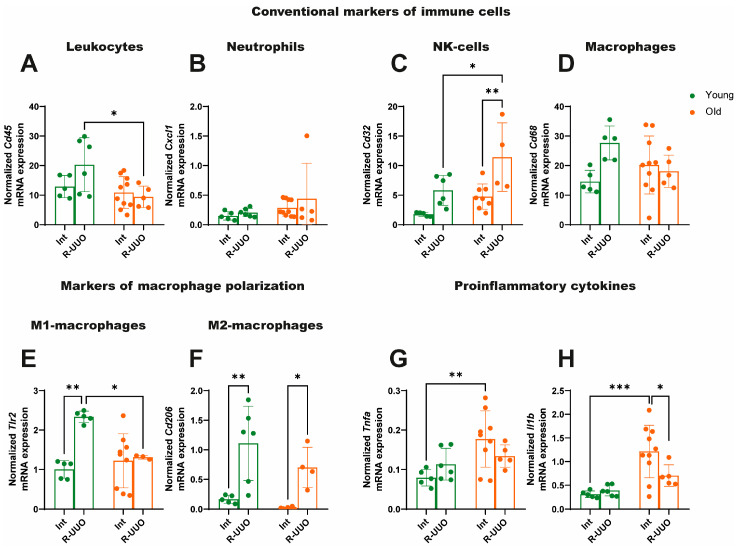
Expression profile of markers of leukocytes (**A**), neutrophils (**B**), macrophages (**C**), NK-cells and monocytes (**D**), markers of M1- (**E**) and M2-macrophages (**F**), and proinflammatory cytokines (**G**,**H**) in young and old rats after R-UUO. The data for young rats are shown in green, and the data for old rats are shown in orange. * *p* < 0.05, ** *p* < 0.01, *** *p* < 0.001 statistically significant differences (two-way ANOVA, Dunnett’s post hoc test). The examples shown are representative of at least 5 independent experiments.

To assess the level of the inflammatory response and the prevalence of different types of immune cells in the damaged kidney following the resolution of obstructive nephropathy, we analyzed the mRNA expression of key pro-inflammatory cytokines, as well as conventional markers of leukocytes, neutrophils, NK cells, and macrophages, including markers of M1 and M2 macrophages. We found that R-UUO induced an increased expression of the leukocyte marker *Cd45* in young rats compared to old ones (Figure 5A), which may indicate more pronounced leukocyte infiltration in the kidney tissue of young animals following ureteral obstruction. While R-UUO had no effect on the expression of the neutrophil marker *Cxcl1* (Figure 5B), it elicited a more significant increase in mRNA expression of *Cd32* (a marker for NK cells and monocytes) in old rats relative to young animals (Figure 5C). The patterns of leukocyte infiltration assessed by *Cd45* were similar to changes in the macrophage marker *Cd68* (Figure 5D), which may indicate the contribution of these cells to the pathogenesis of the immune response in R-UUO. Based on this, we performed analysis of macrophage polarization and revealed a significant upregulation of Toll-like receptor 2 (*Tlr2)*—an M1 macrophage marker mediating pro-inflammatory responses—specifically in young rats following R-UUO; this effect was absent in old animals (Figure 5E). At the same time, the mRNA expression pattern of the M2 macrophage marker *Cd206*, associated with anti-inflammatory responses, was similar between the two age groups (Figure 5F). Notably, expression of the pro-inflammatory cytokines *Tnfa* and *Il1b* was higher in intact old animals than in young ones. However, R-UUO resulted in a decrease in their levels (Figure 5G,H, respectively).

### 3.5. Markers of Autophagy, Oxidative Stress and Mitochondrial Biogenesis

**Figure 6:**
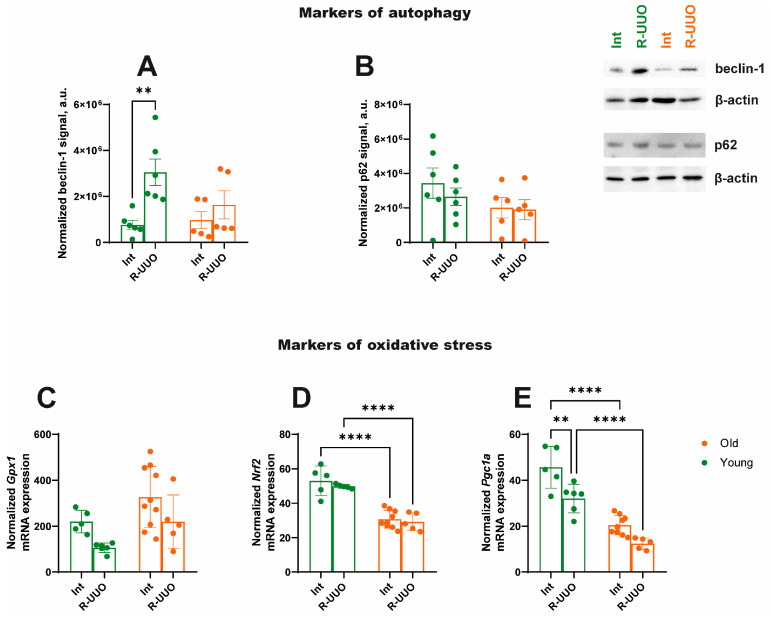
Assessment of autophagic activity, expression profile of markers of oxidative stress and intensity of mitochondrial biogenesis in young and old rats. The level of beclin-1 (**A**) and p62 (**B**) measured by Western blotting in kidneys. Estimation of mRNA expression of *Gpx1* (**C**), *Nrf2* (**D**), and *Pgc1a* (**E**). The data for young rats are shown in green, and the data for old rats are shown in orange. ** *p* < 0.01, **** *p* < 0.0001 statistically significant differences (two-way ANOVA, Dunnett’s post hoc test). The examples shown are representative of at least 5 independent experiments.

To analyze the activation of the autophagy system caused by R-UUO, we assessed the level of beclin-1, which is responsible for autophagosome maturation, as well as the ubiquitin-binding protein p62. R-UUO increased beclin-1 expression in young but not in old animals (Figure 6A). Analysis of the level of the protein p62, which is also associated with autophagy and contains a domain for binding to the autophagosomal protein LC3 [23], tended to decrease in kidney tissue of young rats after R-UUO (Figure 6B), proposing elevated autophagy activation. Regarding markers of oxidative stress, the expression of glutathione peroxidase 1 (*Gpx1*) remained unchanged in response to kidney damage in all experimental groups (Figure 6C), unlike nuclear factor erythroid 2-related factor 2 (*Nrf2*), whose expression decreased in old rats in both experimental groups compared to young rats but was not affected by R-UUO (Figure 6D).

Finally, we observed downregulated expression of the mitochondrial biogenesis marker peroxisome proliferator-activated receptor gamma coactivator 1-alpha (*Pgc1a*) in response to kidney damage in young rats (Figure 6E). In old animals, *Pgc1a* expression was initially reduced compared to young rats and remained unchanged following R-UUO (Figure 6E).

### 3.6. Age-Dependent Mitochondrial Dysfunction Following R-UUO

**Figure 7:**
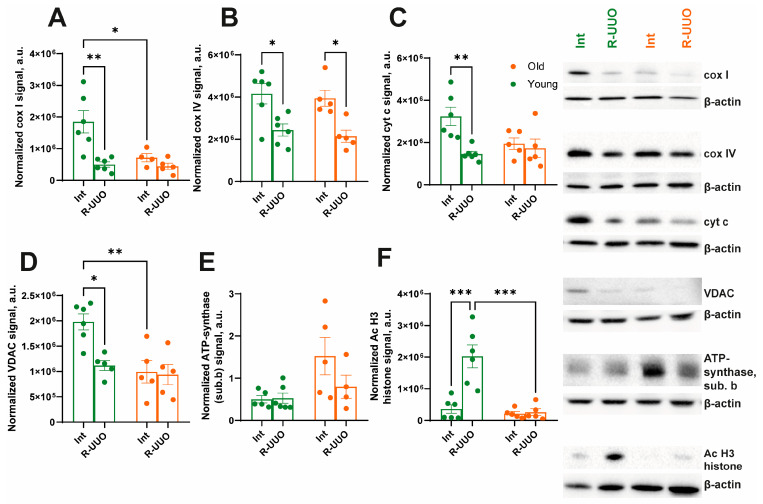
Assessment of the level of key OXPHOS proteins in the young and old kidneys. Protein level of cox I (**A**), cox IV (**B**), cyt c (**C**), VDAC (**D**), ATP synthase subunit b (**E**), and acetylated H3 histone (**F**). The data for young rats are shown in green, and the data for old rats are shown in orange. * *p* < 0.05, ** *p* < 0.01, *** *p* < 0.001 statistically significant differences (two-way ANOVA, Dunnett’s post hoc test). The examples shown are representative of at least 5 independent experiments.

We analyzed age-dependent changes in the levels of key oxidative phosphorylation (OXPHOS) proteins induced by R-UUO. Renal injury induced a pronounced decrease in Complex I (cox I) and IV (cox IV) levels in both age groups (Figure 7A,B, respectively). Additionally, the level of Complex I was lower in intact old rats compared to young ones (Figure 7A). The level of cytochrome c (cyt c) was altered specifically in response to R-UUO, showing a significant decrease in the R-UUO group. No other significant differences in cyt c levels were detected among the experimental groups (Figure 7C). We also assessed VDAC, a mitochondrial outer membrane transporter crucial for metabolism and apoptosis. VDAC expression decreased in young rats following R-UUO, a response that was absent in old animals (Figure 7D). Notably, VDAC expression was already lower in intact old rats compared to young ones (Figure 7D). The level of ATP synthase subunit b remained unchanged across all groups (Figure 7E). Finally, we investigated changes in the acetylation of histone H3 at lysine 9—a modification linked to apoptosis, fibrosis, and mitochondrial dysfunction in kidney diseases [24]. R-UUO triggered a sharp increase in H3 acetylation in young rats, which was not observed in old animals (Figure 7F).

## 4. Discussion

Obstructive nephropathy is one of the common causes of impaired kidney function in patients of different age groups. Depending on the duration of the urinary flow obstruction, it can lead to the development of AKI, CKD, and subsequent tubulointerstitial fibrosis. Age can be an aggravating factor in the development of nephropathy due to the accumulation of pathological changes in cells characterized as a senescent phenotype [25]. We investigated age-dependent changes in renal function, regeneration, and mitochondrial functioning following R-UUO. Reversible obstruction induced a significant impairment of kidney function, as evidenced by elevated SCr and BUN levels in both young and old animals (Figure 2A,B). This pattern of renal dysfunction aligns with established models of AKI, including ischemia-reperfusion [26] and septic injury [27]. At the same time, we observed a more pronounced increase in PCNA levels in response to kidney injury in young animals compared to old ones (Figure 2C). The activation of proliferation in the renal epithelium is a hallmark response to injury, functioning as a dual indicator: it determines the severity of damage and depends on the tissue’s regenerative capacity. Since we observed a higher level of renal dysfunction based on SCr and BUN, the attenuated PCNA response in old rats suggests a reduced kidney regenerative capacity. This may be associated with the accumulation of senescent cells (which are metabolically active but non-dividing) [28], various peptides (e.g., Zag [29]), advanced glycation end products like carboxymethyl-lysine and pentosidine [30], and extracellular matrix proteins [31]. Our study demonstrated that while the expression of key fibrosis markers increased in both young and old rats following R-UUO (Figure 3), young animals exhibited a stronger pro-fibrotic response (Figure 3A–E). These findings are consistent with studies on tubulointerstitial fibrosis progression in terminal UUO models [32,33,34,35]. Indeed, a less pronounced response to injury is a hallmark of aging in experimental models [36,37], often manifesting as impaired wound healing due to reduced collagen turnover and blunted epithelial-mesenchymal transition [38,39].

It is important to acknowledge a limitation inherent to experimental design of our R-UUO model, since we performed a contralateral nephrectomy to assess the functional impairment and regenerative capacity of the post-obstructed kidney. However, the contralateral (unobstructed) kidney could provide compensatory protection against injury caused by obstruction. It was reported, after UUO, the contralateral kidney undergoes functional and structural adaptation: GFR increases, nephrons hypertrophy, and renal plasma flow rises. These compensatory changes help maintain overall renal function [40,41]. Therefore, the pronounced dysfunction and molecular changes we observed might be attenuated in a non-nephrectomized model or in patients with two kidneys. However, the UUO model is widely accepted as a standard model for obstructive nephropathy, as it translates reasonably well to the clinical setting.

Significant tissue damage and concomitant inflammation often induce histological alterations in kidney structure. The observed renal tubule dilation in our experimental groups (Figure 4) is consistent with histological findings from rodent R-UUO models [42,43,44]. For the first time, we have demonstrated the specific features of age-related changes in kidney structure in the context of reversible obstructive nephropathy, such as glomerulosclerosis, focal pericanalicular fibrosis, and lipomatosis (Figure 4). Aged rat kidneys are characterized not only by classic features of age-related nephropathy (glomerulosclerosis and renal tubule fibrosis) but also by lipomatosis—the fatty replacement of functional tissue, most pronounced in the renal sinus [45]. This pathological change may be associated with numerous factors, including inflammation, oxidative stress, and metabolic dysfunction. The accumulation of adipose tissue in the kidney may be a consequence of mitochondrial dysfunction and endoplasmic reticulum stress [46].

Pro- and anti-inflammatory cytokines play a key role in tissue repair, acting as signaling molecules that stimulate the recruitment of immune cells to the site of injury, tissue remodeling, and re-epithelialization. An imbalance in the secretion of these cellular mediators leads to impaired wound healing [47]. Young animals showed a more pronounced increase in the expression of the leukocyte marker *Cd45* following R-UUO than old animals (Figure 5A), which may be a sign of leukocyte infiltration. Furthermore, these changes were consistent with the trends of macrophage marker *Cd68* expression in the kidney tissue (Figure 5D), possibly making these cells key players in the immune response after R-UUO. In contrast, the opposite pattern was observed for the expression of *Cd32* mRNA, a marker for NK cells and monocytes (Figure 5C). Based on these results, we further analyzed the phenotypes of macrophages in the kidney tissue of rats of both ages following R-UUO. We found that expression of the M1-macrophage marker *Tlr2*, which mediates the pro-inflammatory response, increased significantly in the young R-UUO group; this increase was not observed in old animals (Figure 5E). Meanwhile, the dynamics of *Cd206* expression, a marker for M2 macrophages that mediate the anti-inflammatory immune response, were similar in both age groups (Figure 5F). Although age-related changes in macrophage subpopulations are complex, our data suggest impaired M1 macrophage polarization in tissues of old rats. This is supported by studies in aged TLR2-/- mice, which demonstrated that both aging and TLR2 deficiency impair the immune response to Staphylococcus aureus bacteremia. Notably, the development of renal abscesses in this model was more dependent on impaired TLR2 signaling than on age [48]. Furthermore, the age-related decrease in Toll-like receptor expression in macrophages may be linked to an expansion of regulatory T cells, which could collectively suppress immune response activation in aged animals [49,50]. Some studies have reported a higher abundance of M2 macrophages in the bone marrow and muscles of old compared to young animals [51]; these macrophages are also thought to promote angiogenesis following injury [52,53]. Finally, age-related alterations in M1 and M2 macrophage marker expression across experimental groups may reflect shifts in the relative proportions of these subpopulations, as shown in several studies [54,55]. It is crucial to recognize that the inflammatory response to tissue injury is not exclusively detrimental. On the contrary, it is a critical and necessary stage of tissue repair, progressing from acute inflammation through resolution to healing. Dysregulation of this process, whether toward excessive or insufficient inflammation, compromises tissue repair and can promote fibrosis.

Macrophage function declines with age, manifesting as a reduced capacity to phagocytize apoptotic cells and a diminished response to pathogen-associated molecular patterns (PAMPs) in both rodents and humans [56]. This impairment is attributed to deficient phosphorylation of enzymes within macrophage signaling cascades and dysregulated cytokine secretion [57,58]. This defect in secretory function may explain the reduced *Tnfa* and *Il1b* expression observed in aged animals following R-UUO (Figure 5G,H). Furthermore, studies demonstrate impaired TNF-α and IL-1β secretion by peripheral blood mononuclear cells from elderly patients in response to TLR1/TLR2 pathway stimulation [59,60]. Conversely, aging is characterized by a state of chronic inflammation, marked by elevated cytokine levels in tissues [61,62]. This is consistent with the increased mRNA expression of *Tnfa* and *Il1b* observed in aged rats (Figure 5G,H).

Beyond inflammation, autophagy is crucial for renal homeostasis, particularly after injury. Growing evidence indicates that autophagy is pivotal for regulating macrophage polarization [63,64]. Furthermore, autophagy regulates cell senescence, and its dysregulation is implicated in various human diseases, including inflammation, aging, and metabolic disorders [65]. Following R-UUO, young animals exhibited a marked increase in beclin-1 levels compared to aged rats (Figure 6A), suggesting autophagy activation. This is further supported by a concomitant decreasing trend in p62 (Figure 6B), an autophagic substrate degraded with autophagosomes [66]. While consistent with other models [67,68], this is the first assessment of autophagy in aged animals with obstructive nephropathy. Our data reveal an age-related impairment in autophagic degradation of damaged cellular components in the kidney.

An imbalance in the molecular mechanisms governing autophagy, oxidative stress, and inflammation is thought to contribute to the development of both AKI and CKD [69,70]. We found that *Nrf2* expression was reduced in aged rats across experimental groups compared to young rats, though this decrease was not a specific response to R-UUO (Figure 6D). Nrf2 is a key transcription factor regulating the basal and inducible expression of a broad range of antioxidant and xenobiotic-metabolizing enzymes. The Nrf2/ARE pathway represents one of the most critical cellular defense systems against oxidative stress and toxicants [71]. Thus, the observed age-related decline in Nrf2 impairs the renal adaptive antioxidant response, rendering the kidney more vulnerable to oxidative stress, as demonstrated in previous studies [72,73,74].

Oxidative stress is closely linked to mitochondrial function. Mitochondria contribute to the progression of both AKI and CKD through the regulation of redox status and a multitude of other signaling and metabolic pathways [75,76,77]. Analysis of mitochondrial involvement in obstructive nephropathy revealed reduced expression of the mitochondrial biogenesis marker Pgc1a following renal injury in young rats. Notably, *Pgc1a* expression was also lower in intact aged animals compared to young controls (Figure 6E). This finding aligns with observations in kidney biopsies from patients with CKD [78]. Decreased PGC1α levels under UUO conditions serve as an indirect indicator of elevated oxidative stress [79]. Together with the attenuated response to R-UUO, this reduction suggests an impaired antioxidant defense system in aged rats relative to their younger counterparts.

Finally, we identified significant modulation in the expression of mitochondrial respiratory chain protein complexes in both aged and young rats subjected to UUO (Figure 7A–C), which was likely accompanied by impaired respiratory function and ATP synthesis. OXPHOS is frequently impaired during aging and in both acute and chronic kidney diseases, particularly in proximal tubule cells. The reduction in OXPHOS and a metabolic shift toward glycolysis stimulate the development of fibrosis [80,81,82]. An age-related increase in renal cells exhibiting partial loss of respiratory chain cox I and IV has been documented [83,84], reflecting a decline in mitochondrial bioenergetic function [85]. Our study revealed that young rats with R-UUO exhibited a significant decrease in the expression of respiratory chain complexes I and IV, cyt c, and VDAC (Figure 7A–D, respectively), pointing to a substantial impairment of mitochondrial function. Similar findings have been previously reported in studies of irreversible UUO [77].

Furthermore, we demonstrated a sharp increase in histone H3 acetylation at Lys9 in young rats in response to R-UUO, which was not observed in old rats (Figure 7F). Alterations in histone acetylation patterns are associated with changes in the balance of histone acetylases and deacetylases (HDAC) activity, ultimately leading to modulation of the expression of numerous genes involved in cell proliferation, apoptosis, and differentiation in the kidney [86,87]. It has been shown that increased histone acetylation in response to R-UUO may lead to increased transcription of a number of genes associated with the development of inflammation, oxidative stress, and fibrosis [88]. An increase in histone H3 acetylation during ureteral obstruction in young rats may be caused by the activation of NF-kB and the recruitment of HAT-coactivators p300/CBP, which may be confirmed by a decrease in H3 acetylation and TNF-α expression upon inhibition of p300 in the UUO model [89]. At the same time, this mechanism may not work in older animals due to HDAC2 hyperactivation, which blocks p300 recruitment. For example, an increase in HDAC2 in aging tissues has been shown to suppress HAT-dependent transcription [90]. Metabolic regulation by transient reduction of NAD^+^ and inhibition of SIRT1 [91], becomes impossible in old animals due to age-related NAD^+^ depletion and CD38 hyperactivation, as shown in the work on the restoration of epigenetic plasticity with the addition of NAD+ in old mice [92].

Beyond the descriptive analysis of age-dependent signaling pathways, our results open up avenues for potential therapeutic interventions. The marked mitochondrial dysfunction and impaired autophagy observed in aged kidneys after R-UUO make these signaling pathways promising therapeutic targets. For example, strategies aimed at enhancing mitochondrial biogenesis or increasing autophagy flux could be tested for their efficacy in attenuating obstruction-induced damage. Importantly, our data suggest that the response to such interventions is likely to be age-specific. Therapy aimed at attenuating an excessive fibrotic response may be beneficial in young individuals, whereas treatment aimed at restoring bioenergetics and cellular clearance mechanisms may be more appropriate for older kidneys. Evaluating these targeted strategies in preclinical models of obstructive nephropathy is an important next step towards developing age-appropriate therapeutic approaches for clinical practice.

## 5. Conclusions

**Figure 8:**
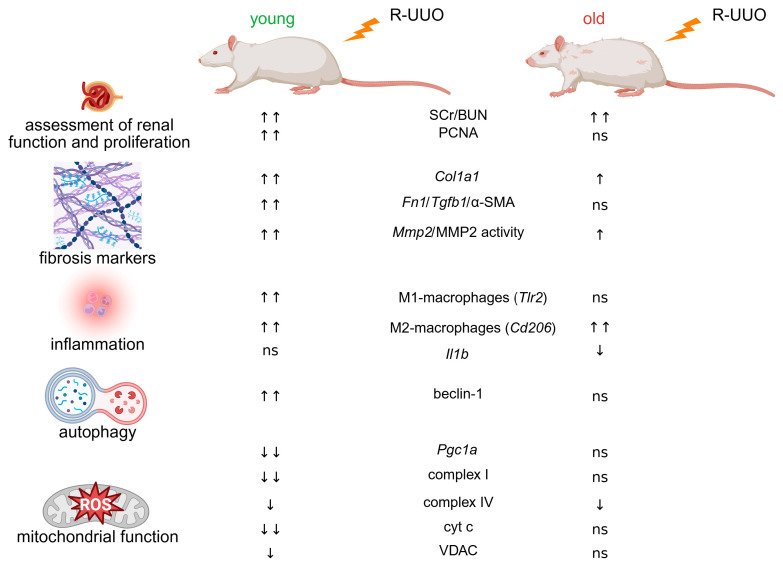
Comparison of kidney tolerance to R-UUO-induced AKI in young and aged rats. Abbreviations: AKI—acute kidney injury; BUN—blood urea nitrogen; Col1a1—collagen type I α1 chain; cyt c—cytochrome c; Fn1—fibronectin1; IL1β—interleukin 1 beta; MMP2—matrix metalloproteinase 2; ns—no significant; PCNA—proliferating cell nuclear antigen; PGC1a—proliferator-activated receptor γ co-activator-1 alpha; SCr—serum creatinine; Tgfb1—transforming growth factor β1; Tlr2—Toll-like receptor 2; R-UUO—reversible unilateral ureteral obstruction; VDAC—voltage-dependent anion-selective channel; α-SMA—alpha-smooth actin. Created in https://BioRender.com (accessed on 7 October 2025).

Comparative analysis of renal tolerance to R-UUO-induced AKI revealed pronounced differences in the inflammatory response, autophagosomal-lysosomal function, and bioenergetic status between young and aged animals (Figure 8). R-UUO impaired ATP production and promotes oxidative stress, inflammation, and autophagy activation. Aging modulated most of these alterations, resulting in an overall maladaptive renal profile in obstructive nephropathy. Our findings highlight the age-related vulnerability to UUO such as diminished epigenetic plasticity, chronic oxidative stress, and impaired autophagy, which may underlie the accelerated progression of obstructive nephropathy in older patients. The study emphasizes that elderly patients with ureteral obstruction may benefit from age-appropriate therapies, as their maladaptive renal responses to obstruction are fundamentally different from those of younger people and are likely to reduce the efficacy of conventional antifibrotic treatments.

## Data Availability

The data that support the findings of this study are available from the corresponding author upon reasonable request.

## Supplementary Materials

The following supporting information can be downloaded at: https://www.mdpi.com/article/10.3390/antiox14101219/s1.

## Abbreviations

The following abbreviations are used in this manuscript:

AKI	Acute kidney injury
BUN	Blood urea nitrogen
CKD	Chronic kidney disease
*Col1a1*	Collagen type I α1 chain
CXCL	C-X-C motif chemokine ligand
cyt c	Cytochrome c
ECM	Extracellular matrix
IL1β	Interleukin 1 beta
GFR	Glomerular filtration rate
*Gpx1*	Glutathione peroxidase 1
MMP	Matrix metalloproteinase
NRF2	Nuclear respiratory factor 2
OXPHOS	Oxidative phosphorylation
PAS	Periodic acid–Schiff staining
PCNA	Proliferating cell nuclear antigen
PGC-1α	Proliferator-activated receptor γ co-activator-1 alpha
p62	Sequestosome 1
SCr	Serum creatinine
TGFβ1	Transforming growth factor β1
TLR2	Toll-like receptor 2
TNFα	Tumor necrosis factor alpha
R-UUO	Reversible unilateral ureteral obstruction
VDAC	Voltage-dependent anion-selective channel
α-SMA	Alpha-smooth actin
