# Supplementary material for: Comparative Analysis of the Tolerance of Young and Old Kidneys to Injury in a Rat Model of Reversible Ureteral Obstruction
Source: Antioxidants (Basel). 2025 Oct 10;14(10):1219. doi: 10.3390/antiox14101219 (PMC12561450; doi:10.3390/antiox14101219)
Supplement: Supplementary file 1 [file antioxidants-14-01219-s001.zip › antioxidants-3884780-supplementary.pdf]

Comparative analysis of the tolerance of young and old kidneys to injury in a rat model of reversible ureteral obstruction

Polina A. Abramicheva, Ilya A. Sokolov, Vasily N. Manskikh, Nadezda V. Andrianova, Dmitry S. Semenovich, Ljubava D. Zorova, Irina B. Pevzner, Egor Y. Plotnikov

Supplementary tables and original images for western blots, zymogram gels and microscopy results

Table S1. Sequences of primers used for gene expression estimation.

Protein	Gene name	Primer nucleotide sequence (5'- to 3')	PCR product size, bp	Genbank accession number
Collagen type I α1 chain	*Col1a1*	for TCAAGATGGTGGCCGTTACT	166	NM_053304.1
		rev CATCTTGAGGTCACGGCATG		
Fibronectin 1	*Fn1*	for TCACAGGGTACAGGATTGT	144	NM_019143.2
		rev TCTCCTCCACAGCATAGATAG		
Transforming growth factor β1	*Tgfb1*	for CGTACACAGCAGTTCTTCTCT	136	NM_021578.2
		rev ATGACATGAACCGACCCTTC		
Matrix metalloproteinase 2	*Mmp2*	for GGACAAGAATCAGATCACATACAG	204	NM_031054.2
		rev TTGCCGTCAAATGGGTATCC		
Interleukin 1β	*Il1b*	for ACACAGGACAGGTATAGATTCTTC	119	NM_031512.2
		rev GGACAGAACATAAGCCAACAAG		
C-X-C Motif Chemokine Ligand 1	*Cxcl1*	for GTGGCAGGGATTCACTTCAAGAAC	170	NM_030845.2
		rev GGGACACCCTTTAGCATCTTTTGG		

Tumor necrosis factor α	*Tnfa*	for CTCCCTCTCATCAGTTCCATGG	296	NM_012675.3
		rev TGGTATGAAATGGCAAATCGGC		
CD45	*Cd45*	for AAGCAATACCACCACAAGCACAG	238	NM_001109890.2
		rev TGGAGTACATGAGCCATTGGAGAG		
CD68	*Cd68*	for TTGAACCCGAACAAAACCAAGGTC	199	NM_001031638.1
		rev GAGAATGTCCACTGTGCTGCTTG		
TLR2	*Tlr2*	for CGGAATGCAGATCTCACCGATA	154	NM_198769.2
		rev TACCTAGCTCCCTCACTACGTC		
CD32	*Cd32*	for TGTCACCATCACTGTCCAAGAG	164	NM_175756.3
		rev CTTCGAGACCAGAAGGAGTGTC		
CD206	*Cd206*	for GATGCAAACCAAACATGCAC	200	NM_001106123.2
		rev ATGTCAGCGTTCCAGTGTG		
Nuclear respiratory factor 2	*Nfe2l2*	for CTCTCTGGAGACGGCCATGACT	146	NM_031789.3
		rev CTGGGCTGGGGACAGTGGTAGT		
Glutathione peroxidase 1	*Gpx1*	for CCTCAAGTATGTCCGACCCG	282	NM_030826.4
		rev TCGATGTCGATGGTGCGAAA		
Pparg coactivator 1 α	*Pgc1a*	for GTGCAGCCAAGACTCTGTATGG	121	NM_031347.1
		rev GTCCAGGTCATTCACATCAAGTTC		

60S acidic ribosomal protein P0	*Rplp0*	for CACAGTACCTGCTCAGAACAC	138	NM_022402.2
		rev ACCTTGTCTCCAGTCTTTATCAG		

Table S2. The number of rats in each experimental group.

Assay	Experimental groups			
	Int young	R-UUO young	Int old	R-UUO old
RT-PCR (kidney)	5	6	10	5
Western blot (kidney)	6	6	5	5
Zymography (kidney)	5	5	5	5
SCr (blood)	6	6	8	5
BUN (blood)	6	6	8	5

Notes: the value of "n" may be less than the number of rats indicated in the table due to the presence of outliers estimated by the ROUT and Grubb's test.

Table S3. Descriptive statistics for all bar graphs.

**Figure**	**Group**	**p value**				**Mean±SD**
		**Int (young) vs R-UUO (young)**	**Int (old) vs R-UUO (old)**	**Int (young) vs Int (old)**	**R-UUO (young) vs R-UUO (old)**	
Fig. 2A	Int young (n=6)	0,0005	<0,0001	0,9969	0,5726	52,67±4,926
	R-UUO young (n=6)					155,3±27,21
	Int old (n=8)					56,75±7,906
	R-UUO old (n=5)					184,4±78,01
Fig. 2B	Int young (n=6)	<0,0001	<0,0001	0,9752	0,5364	6,967±0,5428
	R-UUO young (n=6)					28,75±3,551
	Int old (n=8)					8,038±1,111
	R-UUO					24,82±10,06

	old (n=5)					
Fig. 2C	Int young (n=6)	<0,0001	0,2309	0,988	0,0036	131421±70696
	R-UUO young (n=6)					1641796±521280
	Int old (n=5)					206551±79231
	R-UUO old (n=5)					689602±488674
Fig. 3A	Int young (n=5)	<0,0001	0,0474	0,9874	<0,0001	3,092±0,4040
	R-UUO young (n=6)					34,85±7,821
	Int old (n=10)					2,133±0,8698
	R-UUO old (n=5)					9,888±6,299
Fig. 3B	Int young (n=5)	<0,0001	0,1178	0,9973	0,0012	0,8404±0,2322
	R-UUO young (n=6)					6,675±2,434
	Int old (n=10)					0,6785±0,3751
	R-UUO old (n=5)					2,627±2,204
Fig. 3C	Int young (n=5)	0,011	0,699	0,0487	0,0003	5,193±0,8379
	R-UUO young (n=6)					7,64±1,813
	Int old (n=10)					3,381±0,8327
	R-UUO old (n=5)					4,087±0,9312
Fig. 3D	Int young (n=5)	<0,0001	0,024	0,8349	0,006	1,159±0,3704
	R-UUO young (n=6)					5,464±1,825
	Int old (n=10)					0,3558±0,2086
	R-UUO old (n=6)					2,593±1,928
Fig, 3E	Int young (n=6)	0,0006	0,7187	0,9982	0,0171	131421±70696
	R-UUO					2081101±1172739

	young (n=6)					
	Int old (n=5)					206551±79231
	R-UUO old (n=5)					689602±488647
Fig. 3F	Int young (n=5)	0,0302	0,0416	-	-	77,19±16,65
	R-UUO young (n=5)					107,6±23,65
	Int old (n=5)					92,01±9,65
	R-UUO old (n=5)					122±24,54
Fig. 5A	Int young (n=5)	0,206	0,9691	0,9281	0,0307	12,92±3,735
	R-UUO young (n=6)					20,32±9,132
	Int old (n=10)					10,93±5,363
	R-UUO old (n=5)					9,456±3,636
Fig. 5B	Int young (n=5)	>0,999	0,0003	0,9003	0,0087	0,3074±0,1762
	R-UUO young (n=6)					0,299±0,06619
	Int old (n=10)					0,182±0,1507
	R-UUO old (n=5)					0,7938±0,4355
Fig. 5C	Int young (n=5)	0,0529	0,9543	0,5308	0,2124	14,62±3,834
	R-UUO young (n=6)					32,08±11,96
	Int old (n=10)					20,23±9,81
	R-UUO old (n=5)					18,12±5,452
Fig. 5D	Int young (n=5)	0,1436	0,9106	0,3161	0,0377	1,779±0,3384
	R-UUO young (n=6)					5,819±2,542
	Int old (n=10)					4,765±2,743
	R-UUO					11,4±5,8

	old (n=4)					
Fig. 5E	Int young (n=5)	0,0016	0,9916	0,8425	0,0378	1,004±0,2237
	R-UUO young (n=6)					2,338±0,1436
	Int old (n=9)					1,221±0,6851
	R-UUO old (n=4)					1,312±0,04788
Fig. 5F	Int young (n=5)	0,021	0,0331	0,893	0,3285	0,1644±,06568
	R-UUO young (n=6)					1,109±0,6291
	Int old (n=10)					0,01486±0,01874
	R-UUO old (n=5)					0,7010±0,3421
Fig. 5G	Int young (n=5)	-	-	0,0046	0,7617	0,0792±0,02109
	R-UUO young (n=6)					0,1135±0,04047
	Int old (n=9)					0,1774±0,07148
	R-UUO old (n=5)					0,134±0,02831
Fig. 5H	Int young (n=5)	0,9845	0,2083	0,022	0,9082	0,7598±0,2222
	R-UUO young (n=6)					1,016±0,4038
	Int old (n=10)					2,827±1,819
	R-UUO old (n=5)					1,499±0,4079
Fig. 6A	Int young (n=6)	0,0091	0,7695	0,989	0,1747	767385±481758
	R-UUO young (n=6)					3052723±1402632
	Int old (n=5)					973145±826801
	R-UUO old (n=5)					1637296±1369201
Fig. 6B	Int young (n=6)	0,8223	0,9994	0,4637	0,8606	3440000±2153986
	R-UUO young					2651530±1241997

	(n=6)					
	Int old (n=5)					2016897±1328973
	R-UUO old (n=5)					1902234±1306441
Fig. 6C	Int young (n=5)	0,2733	0,2459	0,2508	0,278	220,5±48,99
	R-UUO young (n=6)					106,5±20,64
	Int old (n=10)					326,6±133,1
	R-UUO old (n=5)					219,8±116,4
Fig. 6D	Int young (n=5)	0,7913	0,9585	<0,0001	<0,0001	53,06±8,559
	R-UUO young (n=6)					49,81±1,157
	Int old (n=10)					30,79±5,166
	R-UUO old (n=5)					29,25±5,131
Fig. 6E	Int young (n=5)	0,0042	0,0898	<0,0001	<0,0001	46,65±9,142
	R-UUO young (n=6)					32,05±6,222
	Int old (n=10)					20,47±4,213
	R-UUO old (n=5)					12,47±2,478
Fig. 7A	Int young (n=6)	0,0013	0,857	0,0144	0,9979	1850463±871464
	R-UUO young (n=6)					497984±205321
	Int old (n=5)					713612±263676
	R-UUO old (n=5)					442945±21981
Fig. 7B	Int young (n=6)	0,0068	0,0215	0,0494	0,9448	4155277±1180098
	R-UUO young (n=6)					2437277±697947
	Int old (n=5)					3944988±843478
	R-UUO old (n=5)					2143486±644370

Fig. 7C	Int young (n=6)	0,058	0,9727	0,0684	0,9413	3238419±1061511
	R-UUO young (n=6)					1456135±327007
	Int old (n=5)					1946633±610694
	R-UUO old (n=5)					1729536±982115
Fig. 7D	Int young (n=6)	0,0112	0,9963	0,0038	0,8926	1980544±386223
	R-UUO young (n=6)					1116801±218646
	Int old (n=5)					992737±493325
	R-UUO old (n=5)					938369±444608
Fig. 7E	Int young (n=6)	0,9999	0,2824	0,058	0,886	2478373±316381
	R-UUO young (n=6)					2536015±1152669
	Int old (n=5)					5064048±3206756
	R-UUO old (n=5)					4006413±2211286
Fig. 7F	Int young (n=6)	0,0001	0,9989	0,9646	0,0001	362190±325826
	R-UUO young (n=6)					2024611±882789
	Int old (n=5)					215823±146732
	R-UUO old (n=5)					262336±243087

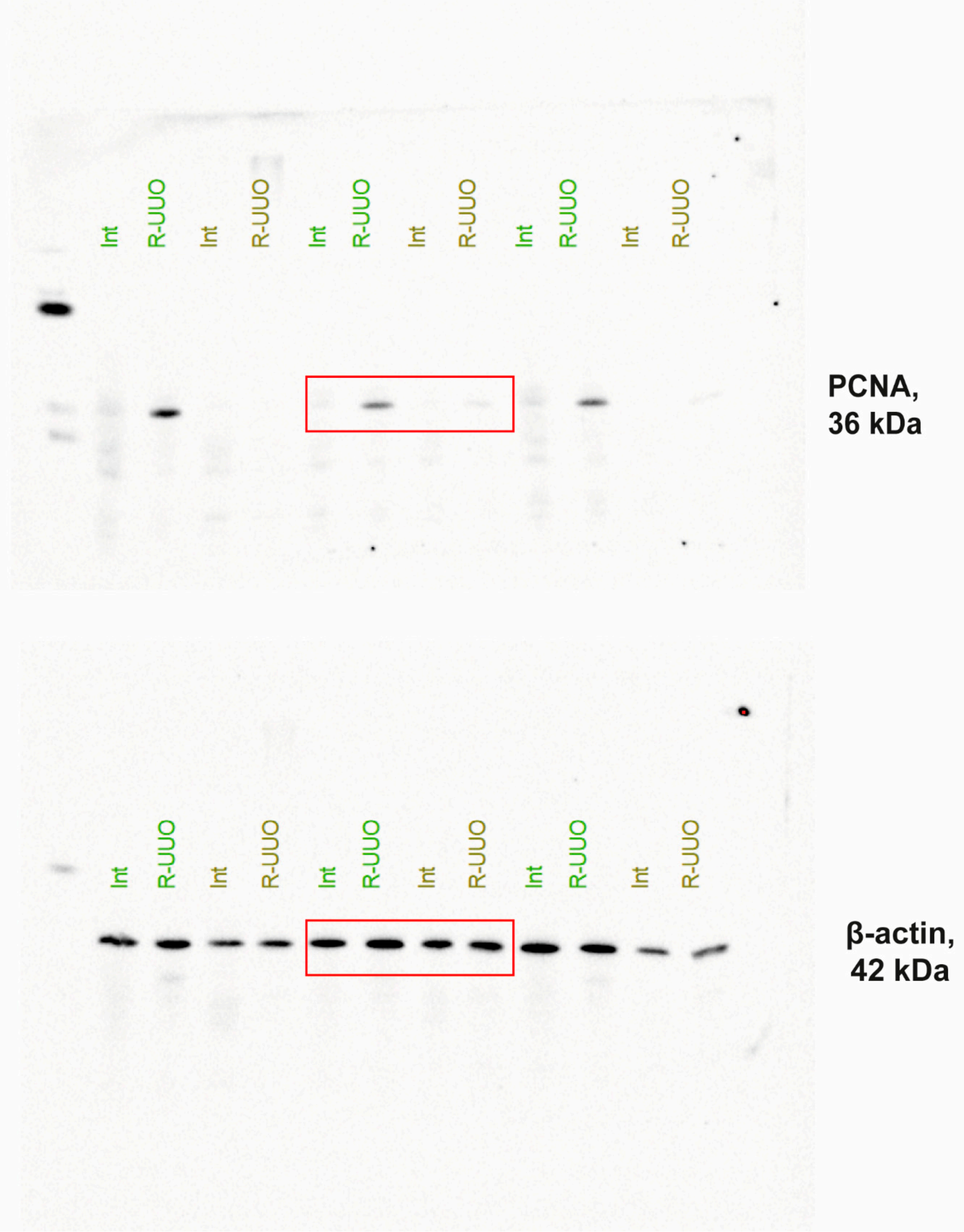

Figure S1. Raw images of western blot of PCNA and β-actin as loading control for PCNA normalization shown in Figure 2.

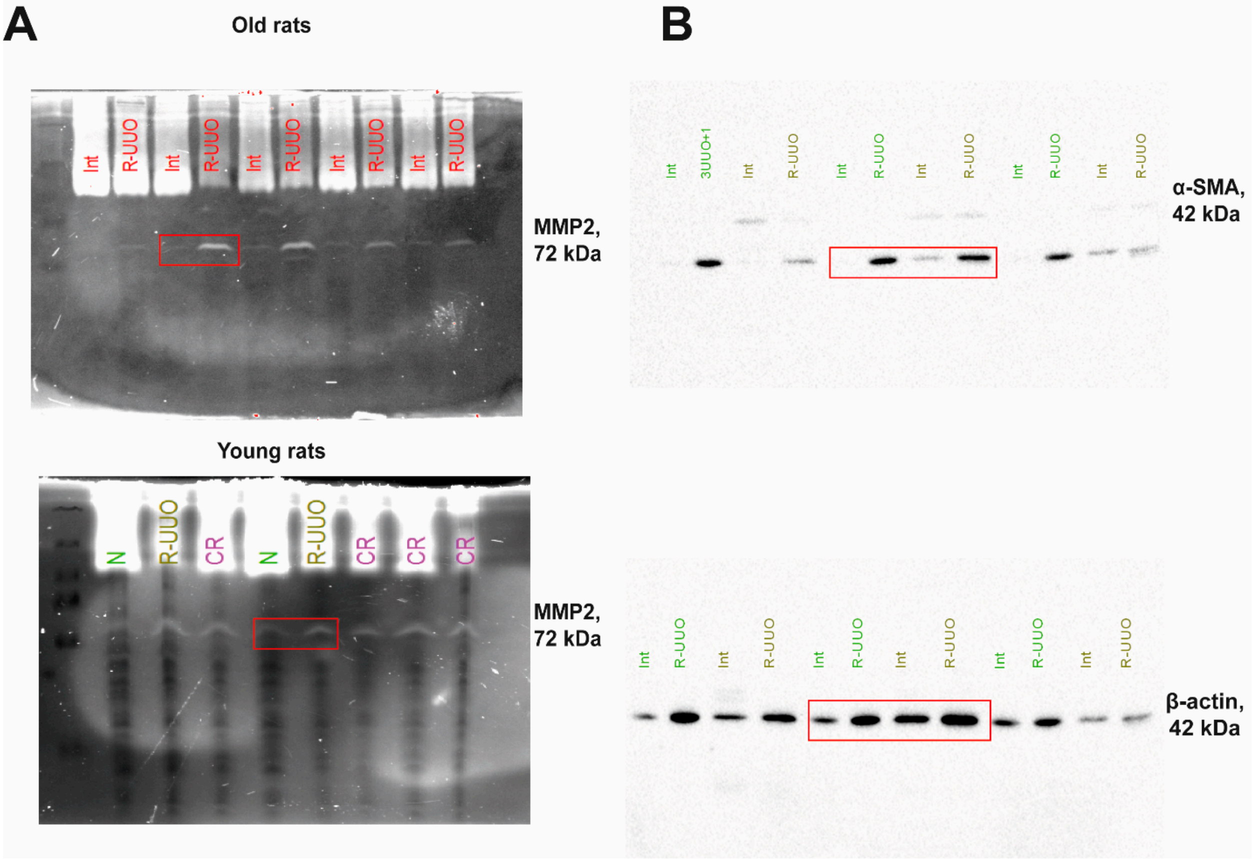

Figure S2. Raw images of zymogram gels (A) and western blot of α-SMA and β-actin as loading control for α-SMA normalization (B) shown in Figure 3.

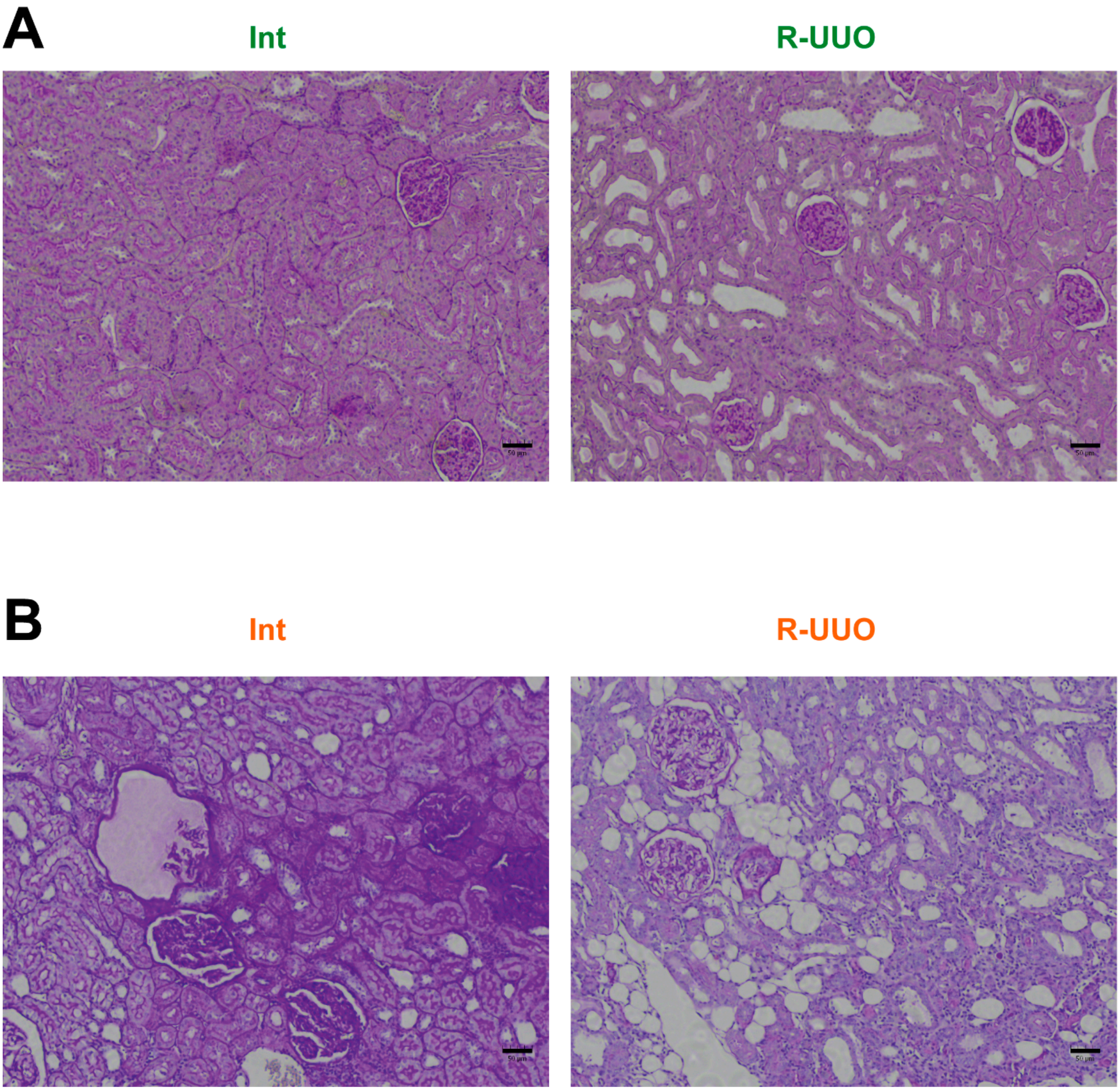

Figure S3. Uncropped images for Figure 4.

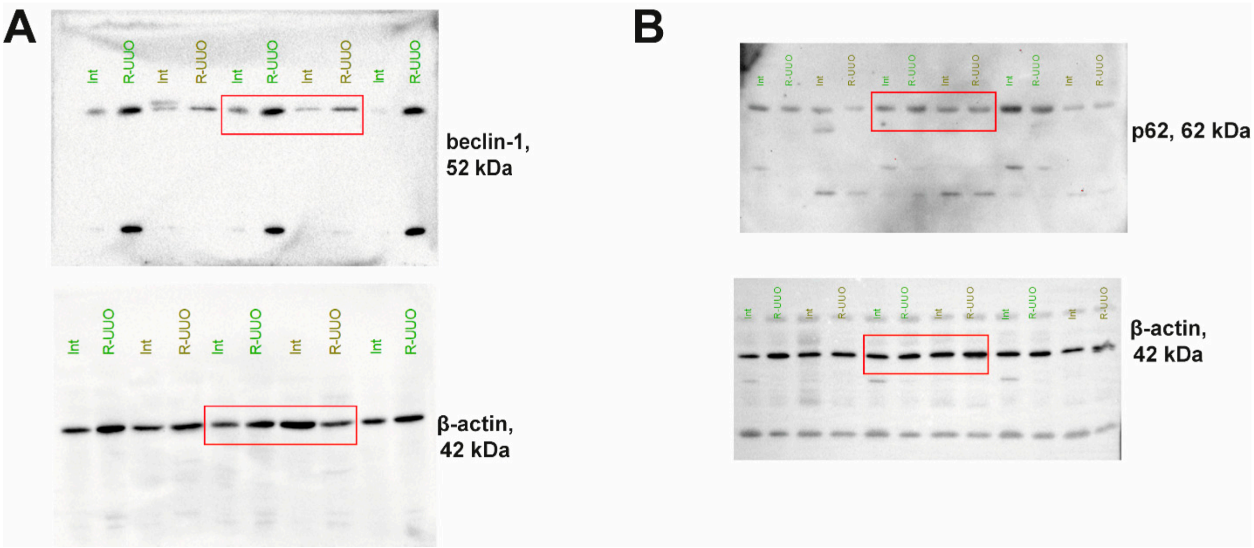

Figure S4. Raw images of western blot of beclin-1 (A), p62 (B), and β-actin as loading control for normalization shown in Figure 6.

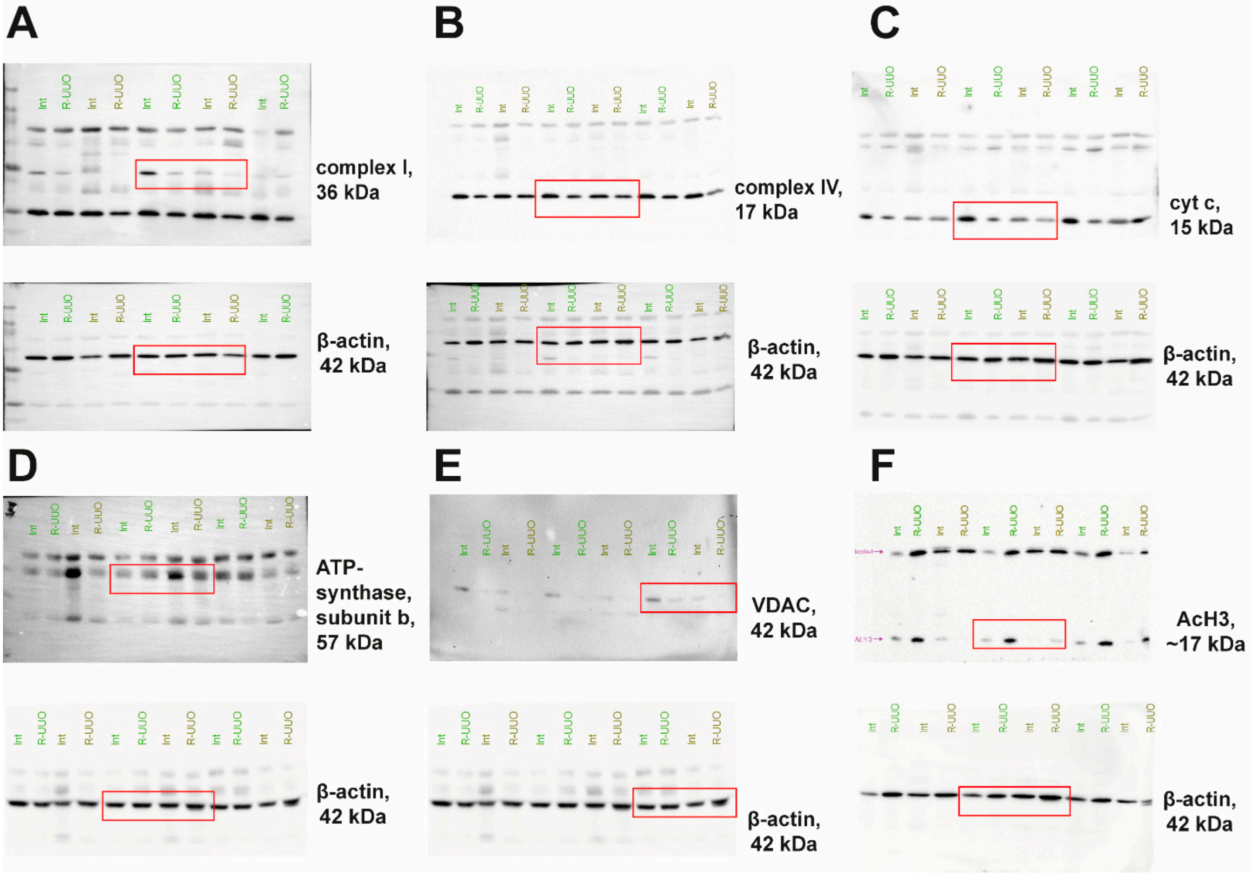

Figure S5. Raw images of western blot of complex I (A), complex IV (B), cyt c (C), ATP-synthase, subunit b (D), VDAC (E) and acetylated histone H3 (F) and β-actin as loading control for normalization shown in Figure 7.
